# Utilization of Genomic Tumor Profiling in Pediatric Liquid Tumors: A Clinical Series

**DOI:** 10.3390/hematolrep15020026

**Published:** 2023-04-19

**Authors:** Ishna Sharma, Min Ji Son, Shoaleh Motamedi, Alice Hoeft, Christa Teller, Tyler Hamby, Anish Ray

**Affiliations:** 1 Texas College of Osteopathic Medicine, The University of North Texas Health Science Center, Fort Worth, TX 76107, USA; 2 Department of Hematology/Oncology, Cook Children’s Medical Center, Fort Worth, TX 76104, USA; 3 Department of Research Operations, Cook Children’s Medical Center, Fort Worth, TX 76104, USA

**Keywords:** leukemia, lymphoma, molecular profiling, targeted therapy, actionable genetic variants

## Abstract

Hematologic tumors are mostly treated with chemotherapies that have poor toxicity profiles. While molecular tumor profiling can expand therapeutic options, our understanding of potential targetable drivers comes from studies of adult liquid tumors, which does not necessarily translate to efficacious treatment in pediatric liquid tumors. There is also no consensus on when profiling should be performed and its use in guiding therapies. We describe a single institution’s experience in integrating profiling for liquid tumors. Pediatric patients diagnosed with leukemia or lymphoma and who underwent tumor profiling were retrospectively reviewed. Ten (83.3%) patients had relapsed disease prior to tumor profiling. Eleven (91.7%) patients had targetable alterations identified on profiling, and three (25%) received targeted therapy based on these variants. Of the three patients that received targeted therapy, two (66.7%) were living, and one (33.3%) decreased. For a portion of our relapsing and/or treatment-refractory patients, genetic profiling was feasible and useful in tailoring therapy to obtain stable or remission states. Practitioners may hesitate to deviate from the ‘standard of therapy’, resulting in the underutilization of profiling results. Prospective studies should identify actionable genetic variants found more frequently in pediatric liquid tumors and explore the benefits of proactive tumor profiling prior to the first relapse.

## 1. Introduction 

Malignant neoplasms are the third leading cause of death among pediatric patients [1]. Unlike adults, children have higher incidence rates of liquid cancers than solid cancers [1,2]. Liquid cancers are malignancies arising from bone marrow cells or lymph nodes and include leukemias and lymphomas. Acute lymphoblastic leukemia (ALL), followed by acute myeloid leukemia (AML), is the most common leukemia found in children [2,3]. 

Over the past 60 years, five-year survival rates for pediatric liquid tumor patients have increased from 10% to 80% due to advancements in conventional chemotherapy treatments and supportive care [2,3]. Yet, for those patients with relapsing and/or refractory liquid cancers, prognosis and quality of life are dismal: reinduction treatment for such cancers relies heavily on escalating the intensity of chemotherapy drugs. Such treatment regimens are limited by short-term and long-term treatment toxicities and/or reduced efficacy [4,5,6]. 

To overcome similar treatment hurdles in solid adult cancers, precision medicine (i.e., targeted therapy) is often utilized to target particular enzymes and/or signal transducers involved with tumor growth [7]. With a better understanding of the genetics underlying tumor prognosis, molecular profiling use has expanded to adult liquid tumors to guide therapeutic options. 

Unfortunately, the application of targeted therapy to pediatric liquid tumors is generally limited; frequently, the therapies are developed to target oncogenic mutations that occur in adult liquid tumor counterparts. Despite similar cells of origin, pediatric liquid tumors often have different oncogenic drivers than those in adults. This frequently means that the targeted therapies developed to treat adult liquid tumors have lower efficacy for treating pediatric liquid tumors [8]. As an example, an analysis of the pediatric AML molecular landscape found that mutations in the Muscleblind Like Splicing Regulator (*MBNL1*), Zinc Finger E-Box Binding Homeobox 2 (*ZEB2*), and E74-like factor 1 (*ELF1*) genes were disproportionately prevalent in pediatric AML tumors in comparison to adult AML tumors [9]. Further, mutations in DNA methyltransferase 3 alpha (*DNMT3A*) and tumor protein 53 (*TP53*) genes, which drive AML in adult patients, were absent in nearly all pediatric AML cases analyzed [9,10,11]. 

Additionally, there has also been limited use of molecular profiling beyond the basic classification of leukemias and lymphomas in children. The College of American Pathologists and the American Society of Hematology recently acknowledged that pediatric ALL is more heterogeneous than previously understood, especially for relapsing and refractory patients. As such, the bodies recommended the use of molecular profiling in identifying biomarkers, prognostic factors, and genetic abnormalities for therapeutic targets [12,13]. There remains, however, limited consensus on how and when to use molecular profiling to guide treatment options for pediatric liquid tumors. Molecular profiling is more heavily used in relapsing or refractory pediatric liquid cancers once other options have been exhausted, but it is not generally considered part of first-line treatment plans or during initial relapse/refractory episodes [14,15]. 

In this study, we aim to present a single institution’s experience with integrating molecular profiling to guide diagnoses and therapies for pediatric patients with leukemias or lymphomas. We describe the clinical benefits (e.g., potentially actionable variants) of liquid tumor genetic profiling and molecular tumor boards to identify the utility of the profiling results. 

## 2. Methods

A retrospective study was performed on patients with leukemia or lymphoma diagnoses between April 2011 and August 2021; the diagnosis was determined via bone marrow biopsy or liquid cytology. Included patients also underwent tumor genomic profiling by Foundation Medicine (Cambridge, MA, USA) during the course of treatment. Only patients <21 years of age were included in this study. Patients were excluded if profiling yielded ‘undetermined’ results. The primary study endpoint was upon patient death or 1 December 2021, whichever came earlier.

### 2.1. Data Collection

Data was collected from queries of the electronic medical record system and stored in the REDCap database. The data that were pulled for patients meeting eligibility criteria for this study included cancer diagnosis, age at the time of diagnosis, gender, ethnicity/race, and tumor profiling results. The clinical course was also documented, including therapies utilized, the influence of treatment plans based on genetic profiling reports, the number of therapies attempted, and clinical outcomes. Outcomes were further subdivided as responses to therapies, including remission, relapse, progression, and mortality. 

### 2.2. FoundationOne Testing

Tumor samples were sent to Foundation Medicine, Inc for genetic sequencing. Samples were sent once patients experienced relapse and/or demonstrated treatment resistance except for two patients with known aggressive disease presentation; the aggressive presentation was dictated by clinical presentation and/or limited initial molecular testing performed on all leukemia patients at our institution to identify common prognostic biomarkers (e.g., *BCR-ABL*). The FoundationOne Heme (F1H) test was used for these liquid tumors, which is a next-generation sequencing in vitro assay that detects four primary genomic alteration classes: base substitutions, copy number alterations, insertions and deletions, and rearrangements. 

Results also included biomarker analysis, such as microsatellite status (MS) and tumor mutation burden (TMB). MS was categorized as stable or unstable; TMB was characterized as low (1–5 mutations/mb), intermediate (6–19 mutations/mb), or high (>19 mutations/mb). Reports also included remarkable genomic alterations for the cancer type (variants of significance), variants of unknown significance, and matched therapies in the form of Federal Drug Administration-approved therapies, clinical trials, and/or off-label therapies. 

Additionally, whether therapy recommendations from the report were incorporated into patient care were captured. Patients were categorized into mutually exclusive groups based on their identified variants: patients who did not have any potentially actionable variants were considered “un-matched”, and patients with identified potentially actionable variants were considered “matched. Of the “matched” patients, those who received targeted therapy related to the identified variant were considered “matched-received” and those that did not were considered “matched-not received”. 

### 2.3. Statistical Analyses

Standard descriptive analysis was used to explain the characteristics of the study population, diagnoses, treatment, and outcome variables. Analyses included using medians and ranges for continuous variables; and frequencies and percentages for categorical variables.

## 3. Results 

A total of 12 patients met the inclusion criteria. The median (range) age at the time of liquid tumor diagnosis was 6.6 (1.0–18.9) years. There were 7 (58.3%) males and 5 (41.7%) females. Three (25.0%) patients identified as white, non-Hispanic and 9 (75.0%) identified as Hispanic. Leukemia was the predominant cancer diagnosis in our cohort, with 5 (41.7%) patients diagnosed with ALL and 6 (50.0%) diagnosed with AML; 1 (8.3%) patient was diagnosed with anaplastic large cell lymphoma (Table 1). 

From the tumor profiling reports, a total of 113 genetic variants were identified across the cohort.

Twenty-eight (24.8%) variants were found to be of significance but not matched to targeted therapy. Seventy-eight (69.0%) of the genetic variants were of unknown significance. While all 12 (100.0%) patients had one or more variants of significance, only 8 (66.7%) were matched to a targeted therapy based on the variant; of these 8, 3 (37.5%) patients received targeted therapy based on the identified targetable variations. 

Three (25.0%) patients had an unknown MS status, and 9 (75.0%) had a stable status. Four (33.3%) patients had an unknown TMB, and 8 (66.7%) had a low TMB. Prior to tumor genetic profiling, 10 (83.3%) had relapsed disease, of which 3 (30.0%) had initial refractory disease. At the time of the study conclusion, 6 (50.0%) patients were alive, and 6 (50.0%) were deceased (Table 2).

### 3.1. Acute Lymphoblastic Leukemia (ALL)

A total of five patients were diagnosed with ALL. Of these five patients, three (60.0%) were male, and two (40.0%) were female. The median (range) age at diagnosis was 10.1 (1.4–18.9). For all patients, profiling was performed after established relapse and/or treatment resistance. Of these patients, three (60%) had targetable variants, and two (40%) had unknown variants. At the time of review, three (60%) patients were deceased, while the two (40%) living patients had each relapsed three times. 

The most commonly identified genetic variant of significance involved the *CDK2NA/B* genetic locus, which was seen in 3 (60%) patients. There is no known targetable therapy identified for this variant. Further, neither this nor any other variant presented at a statistically significant rate among ALL patients. 

Additional targetable genetic variants included *ABL1* (1 patient) and *KRAS* (1 patient) genetic loci, which were matched to Dasatinib during profiling and were subsequently administered (Table 1). Targeted therapy approved for the *ABL1* genetic variant includes Bosutinib, Dasatinib, Imatinib, Nilotinib, and Ponatinib (e.g., tyrosine-kinase inhibition). See Supplementary Material for a comprehensive list of identified genetic variants (Table S1) and targetable variants (Table S2). 

### 3.2. Acute Myeloid Leukemia (AML)

A total of 6 patients, 3 (50.0%) females and 3 (50.0%) males, were diagnosed with AML. The median (range) age at the time of diagnosis was 7.9 (1.7–17.9) years. For one patient (16.7%), profiling was performed prior to relapse due to the high-risk nature of the disease; the remaining were profiled after the establishment of relapse and/or treatment resistance. For 1 (16.7%) patient, therapy included precision medicine identified by the tumor profiling report. In particular, the patient presented with a KRAS genetic loci and was treated with trametinib, a mitogen-activated extracellular kinase (MEK) inhibitor (Table 1). 

For the remaining patients, analysis for one (16.7%) patient revealed an *ATM* (ataxia telangiectasia) variant, targetable with Olaparib, Rucaparib, and Niraparib (e.g., BCL2 and pro-apoptotic pathway mechanisms), and an *ABL1* variant, targetable with Bosutinib, Dasatinib, Imatinib, Nilotinib, and/or Ponatinib (e.g., *BCR-ABL* kinase pathway mechanisms). One patient presented with *CSF3R T618I* genetic variant targetable via the JAK-STAT pathway, one patient with a *BRCA2* and *NPM1-ALK* fusion genetic variant, and one patient without variants of significance. See Supplementary Material for a comprehensive list of identified genetic variants (Table S1) and targetable variants (Table S2). 

These four patients were not treated with targeted therapy identified by the report, although therapy for at least one patient involved targeted therapy, such as sorafenib. Among the AML patients, there was no genetic variant that presented at a statistically significant rate, although *BLM*, *PTPN11*, and *MED12* variants were present in more than one patient. At the time of the study conclusion, three (50.0%) patients were deceased, while 3 (50.0%) patients were alive and in remission. 

### 3.3. Lymphoma

One (8.3%) male patient was diagnosed with anaplastic large-cell lymphoma. Identified genetic variants of significance included *NPM1-ALK* fusion, *PIM1* amplification, and *STAG2* rearrangement exon 5. Identified targetable treatments focused on *NPM1-ALK* fusion; therapies included a clinical trial of Alectinib, Brigatinib, Ceritinib, and/or Crizotinib (Table 1). The clinical course involved two relapses and one clinical trial prior to genetic tumor profiling, after which treatment included the matched therapies noted above. The patient was living at the conclusion of this study.

## 4. Discussion 

Among the genetic variants reported, no clear pattern was seen with regard to a dominant biomarker(s) and/or mutation(s). Common mutations found in leukemia patients, such as *ABL1*, *FGFR1*, *KRAS*, *MET*, *NOTCH1*, and *PTPN11*, were also found among our patient population, although not universally. [16] The literature notes that pediatric leukemias have more genetic heterogeneity than previously understood [17,18]. Thus, the lack of a common variant or set of particular variants among our relapsing and/or refractory ALL and AML patients is unsurprising.

Further, a pan-cancer analysis of pediatric liquid tumors found that 78 of 142 driver genes were not found in adult pan-cancer studies, suggesting that the evolution of counterpart cancers is due to different causes, some of which may not be detected with current modes of profiling [19]. 

Additionally, despite the identification of matched therapies for a majority of our ALL and AML patients, a minority (25.0%) of patient therapies were guided by the results of genetic profiling. It highlights, among other things, the hesitation that practitioners have in deviating from the ‘gold standard for therapy’ and the lack of literature that supports the use of these drugs in ALL or AML treatment. 

### 4.1. Clinical Impact of Molecular Profiling in Acute Lymphoblastic Leukemia (ALL)

ALL is the most common type of childhood cancer and has a favorable prognosis with a 5-year survival rate exceeding 85% [20]. With relapse and refractory disease, however, survival rates decline significantly due in part to increasing drug resistance. Aggressive therapy is the most effective treatment for relapse; Pierro et al. note the importance of detecting specific markers associated with relapse early in the course of treatment to target pathways that lead to a decrease in relapse risk [21]. 

Indeed, performing genetic somatic sequencing panels for pediatric cancers has been shown to significantly impact clinical care in 78.7% of pediatric patients [21]. Further, early inclusion of targeted therapy reduces the risk of relapse, improves disease-free progression, and improves overall outcomes [21]. 

Although early detection of genetic variants and inclusion of targeted therapies are important, these techniques have low utility if genetic panels are not tailored to mutations present in children. Among adult ALL patients, the most common mutations are kinase-activating fusions (90% of ALL adults), MLL gene rearrangement (10% of all ALL adults), and Notch signaling pathway mutations (10% of ALL adults) [22,23]. In pediatric patients, however, these mutations constitute 8.9%, 5%, and 60+% of ALL cases, respectively [22,23,24,25]. In contrast, relapsed ALL pediatric patients have been found to have a variety of mutations in *N-RAS, KRAS*, 5′-nucleotidase, cytosolic II (*NT5C2*), and phosphoribosyl pyrophosphate synthetase 1 (*PRPS1)* [26]. These mutations may not be commonly included in tumor profiling developed on adult ALL variants, and thus, early detection may be missed. This may delay proactive treatment adjustments, such as aggressive inclusion of thiopurine for *NT5C2* and *PRPS1* mutations during initial treatment phases [26]. This may also explain the lack of identified targetable variants among our ALL population. 

### 4.2. Clinical Impact of Molecular Profiling in Acute Myeloid Leukemia (AML)

Treatment for pediatric AML experienced similar issues as in pediatric ALL. In particular, differences in oncogenic drivers between adult and pediatric AML can lower the detectability of critical driver mutations and, thus, the inclusion of efficacious targeted therapy. As previously mentioned, the Muscleblind Like Splicing Regulator (*MBNL1*), Zinc Finger E-Box Binding Homeobox 2 (*ZEB2)*, *MLL* gene, and E74-like factor 1 (*ELF1*) genes are disproportionately prevalent in pediatric AML tumors in comparison to adult AML tumors [9,26]. Further, mutations in DNA methyltransferase 3 alpha (*DNMT3A*) and tumor protein 53 (*TP53*) genes, which drive AML in adult patients, are absent in most pediatric AML cases [10,11,12]. 

Among our cohort, one patient had an FMS-like tyrosine kinase 3 (*FLT3*) mutation. Nearly 30% of pediatric AML cases present with this mutation and are associated with poorer outcomes due to increased resistance to tyrosine kinase inhibitor therapy [27]. These patients can be treated with Gilteritinib, an *FLT3* inhibitor used for refractory AML cases. However, additional mutations in the *FLT3* genetic loci (e.g., *FLT3-F691L*) can cause resistance to gilteritinib, as well as midostaurin, quizartinib, sorafenib, and crenolanib [28]. Further, co-occurring mutations, such as *KRAS* in our case, increase the risk for refractory AML and gilteritinib resistance [29]. Thus, it is useful to determine mutation relations that decrease targeted therapy efficacy early on, as well as how prevalent such groups of mutations are in the pediatric liquid tumor population versus the adult liquid tumor population. 

Of additional interest are the neurotrophic receptor tyrosine kinases (*NTRKs*), nucleophosmin 1 (*NPM1*), and the *CSF3R* mutations, which are present to a greater degree in pediatric liquid tumors than previously thought [30,31,32,33,34]. 

### 4.3. Sequencing and Report Interpretation 

Next-generation somatic sequencing panels are generally designed to detect common mutations found in adult cancers, which may or may not extend to pediatric hematologic malignancies [35]. Newman et al. report that the sequencing test FoundationOne Heme, as used in this study, outperformed other commonly used panels, covering 84% of reported variants in pediatric liquid tumors; however, a combination of whole-genome sequencing, whole-exome sequencing and RNA sequencing provided better identification of genetic drivers [36]. Existing literature further notes that despite the extensive genetic coverage of sequencing panels, they have relatively limited use in the pediatric cancer population [37,38,39,40,41,42,43]. Panels tailored to mutations driving pediatric hematologic cancers would not only close the gap in identifying potentially targetable mutations, including in patients presenting with aggressive cancers, but also better match therapies for improved clinical outcomes. 

Further, the timing for initial and repeat orders of these genetic tests remains uncertain. Treatment and/or the natural course of malignant pediatric hematopoietic cancer cells often leads to enhanced accumulation of mutations, warranting repeat genetic testing as therapy progresses [44]. We speculate that testing for patients at the first sign of relapse or resistance would be beneficial in understanding mechanisms underlying tumor growth, as well as in avoiding inappropriate therapies. Furthermore, expanded testing for pediatric leukemias and lymphomas at diagnosis may allow for the incorporation of targeted therapies early on, as opposed to high-dose chemotherapies that result in short- and long-term toxicities. Current recommendations, while suggesting the importance of molecular profiling in liquid tumors, do not recommend standard profiling for at least a subset of tumors, unlike in adult patients [12,13]. However, our experience and other studies have demonstrated the feasibility of incorporating sequencing in pediatric cancer care [36,44]. 

As tumor profiling encompasses more genetic variants and becomes more widespread, multidisciplinary molecular tumor boards would be beneficial in determining the utility of reported oncogenic drivers and matched therapies. With physicians, pharmacists, scientists, and bioinformaticians working together, it’s possible to efficiently identify targetable mutations, determine which targeted therapies work best for the presenting tumor type and validate the feasibility of carrying out treatment. Collaborative tumor boards also aid in identifying mutation combinations that may otherwise be overlooked but are critical to rule in or out certain targeted therapies, as was the case in our cohort. 

### 4.4. Study Limitations

While this study provides insight into the potential utility of tumor molecular profiling for liquid tumors, we recognize several limitations. Since this was conceived as a single-institution study, the patient population was relatively limited in diagnosis representation and number. As such, it was difficult to define a prevalent mutation in pediatric liquid tumors; a larger prospective study that employs genomic profiling at the time of diagnosis, remission, and relapse would be beneficial in investigating the use of tumor profiling. Further, variation in clinician practice made it difficult to definitely declare that identified targeted therapies or tumor profiling attributed to clinical course and outcomes. As mentioned above, it is not standard clinical practice to conduct tumor profiling for liquid tumors unless the patient relapses or is treatment-resistant; in such instances, profiling may still not be conducted, limiting data on potentially actionable genetic variations. Despite this, the study provides a description elucidating the importance of genomic profiling in guiding therapies for pediatric liquid neoplasms and the feasibility of incorporating profiling in early/multiple stages of diagnosis and treatment. 

## 5. Conclusions

The use of genetic tumor profiling has increased rapidly in the past decade as it can broaden therapeutic options. For liquid pediatric tumors, tumor profiling remains underutilized due in part to a lack of pediatric-focused sequencing, lack of oncogenic driver knowledge, and lack of consensus on when to incorporate profiling during care. In our study, we note that these very hurdles prevented greater use of the profiling studies to tailor therapies for our relapsed and/or refractory patients. As such, for liquid pediatric tumors, prospective studies investigating oncogenic drivers specific to pediatric patients, as well as the development of sequencing panels specific to these drivers, would prove beneficial. Moreover, widespread use of these tests for all pediatric liquid tumor patients, not only those that have relapsed or refractory disease, would aid in successfully guiding clinical decisions while reducing therapy toxicity. 

## Figures and Tables

**Table 1:** Patient Demographic and Diagnoses. PD = progressive disease; SD = stable disease.

Age at Diagnosis (Years)	Diagnosis	Microsatellite Stability Status	Tumor Mutation Burdens	Identified Variant that Guided Treatment	Targeted Therapy Received Based on Identified Variant	Best Clinical Outcome	Patient Status
1.67	Mixed Phenotype Acute Leukemia (MPAL)	Stable	Low (2)	-	-	SD	Alive
1.04	Acute Megakaryoblastic Leukemia (M7 AML)	Stable	Low (2)	-	-	PD	Deceased
4.6	Acute Myeloid Leukemia	Unknown	Low (3)	-	-	SD	Alive
8.56	Acute Megakaryoblastic Leukemia (M7 AML)	Stable	Unknown	-	-	PD	Deceased
13.59	Acute Myeloid Leukemia	Stable	Low (1)	-	-	SD	Alive
17.93	Acute Myeloid Leukemia	Stable	Low (0)	KRAS	Trametinib	PD	Deceased
1.35	B-cell Acute Lymphoblastic Leukemia	Unknown	Unknown	-	-	SD	Alive
3.98	B-cell Acute Lymphoblastic Leukemia	Stable	Unknown	ABL1	Dasatinib	SD	Alive
9.28	T-cell Acute Lymphoblastic Leukemia	Stable	Unknown	KRAS	-	PD	Deceased
18.93	Pre-B-cell Acute Lymphoblastic Leukemia	Stable	Low (2)	-	-	PD	Deceased
16.88	B-cell Acute Lymphoblastic Leukemia	Stable	Low (2)	-	-	PD	Deceased
2.51	Anaplastic Large Cell Lymphoma	Unknown	Low (2)	NPM1-ALK Fusion	Crizotinib, as part of the ANHL12P1 clinical trial	SD	Alive

**Table 2:** Stratification of patients into targetable genetic match groups.

Variable	Results	Avg Prior Relapses	Avg Prior Lines of Therapy	Total N = 12	Matched-Not Received N = 8	Matched-Received N = 3	Unmatched N=1
Diagnosis	Acute Myeloid Leukemia	1	1.7	6	5	1	0
	Acute Lymphoblastic Leukemia	1.6	1	5	3	1	1
	Anaplastic Large Cell Lymphoma	2	2	1	0	1	0

## Data Availability

Not applicable.

## Supplementary Materials

The following supporting information can be downloaded at: https://www.mdpi.com/article/10.3390/hematolrep15020026/s1, Table S1: Comprehensive index of genetic variants identified with liquid tumor profiling; Table S2: Comprehensive list of identified targetable genetic mutations.
