# Supplementary material for: Utilization of Genomic Tumor Profiling in Pediatric Liquid Tumors: A Clinical Series
Source: Hematol Rep. 2023 Apr 19;15(2):256–65. doi: 10.3390/hematolrep15020026 (PMC10123750; doi:10.3390/hematolrep15020026)
Supplement: Supplementary file 1 [file hematolrep-15-00026-s001.zip › hematolrep-2030581-supplementary.pdf]

Table S1. Comprehensive index of genetic variants identified with liquid tumor genetic profiling, including those of significance, of targetable significance, and of unknown significance. *Patients (ALL, AML, Lymphoma)

Alterations	Total (N=12)	Acute Lymphoblastic Leukemia (N=5)	Acute Myeloid Leukemia (N=6)	Anaplastic Large Cell Lymphoma (N=1)
220+1G>T	1 (0, 0, 1)		1 (0, 0, 1)	
ABL1	2 (0, 2, 0)	1 (0, 1, 0)	1 (0, 1, 0)	
ATM	1 (0, 1, 0)		1 (0, 1, 0)	
ATR	1 (0, 0, 1)	1 (0, 0, 1)		
ATRX	1 (0, 0, 1)		1 (0, 0, 1)	
AURKB	1 (0, 0, 1)	1 (0, 0, 1)		
AXIN1	1 (0, 0, 1)	1 (0, 0, 1)		
B2M	1 (0, 0, 1)	1 (0, 0, 1)		
BCL11B	1 (0, 0, 1)	1 (0, 0, 1)		
BLM	3 (1, 0, 2)	1 (0, 0, 1)	2 (1, 0, 1)	
BLM C11ORF30 (EMSY)	1 (1, 0, 0)		1 (1, 0, 0)	
BRCA2	2 (0, 0, 2)	1 (0, 0, 1)	1 (0, 0, 1)	
BTG1	1 (0, 0, 1)	1 (0, 0, 1)		
CD22	1 (1, 0, 0)		1 (1, 0, 0)	

Alterations	Total (N=12)	Acute Lymphoblastic Leukemia (N=5)	Acute Myeloid Leukemia (N=6)	Anaplastic Large Cell Lymphoma (N=1)
CDKN2A	4 (0, 0, 4)	3 (0, 0, 3)	1 (0, 0, 1)	
CIITA	1 (0, 0, 1)	1 (0, 0, 1)		
CSF3R	1 (0, 1, 0)		1 (0, 1, 0)	
CTCF	1 (0, 0, 1)	1 (0, 0, 1)		
CTNNA1	1 (0, 0, 1)	1 (0, 0, 1)		
CUX1	1 (0, 0, 1)	1 (0, 0, 1)		
DOT1	1 (0, 0, 1)	1 (0, 0, 1)		
ERBB4	1 (1, 0, 0)		1 (1, 0, 0)	
ETV6	1 (0, 0, 1)	1 (0, 0, 1)		
FANCD2	1 (0, 0, 1)	1 (0, 0, 1)		
FGF14	1 (1, 0, 0)		1 (1, 0, 0)	
FGFR1	1 (0, 0, 1)	1 (0, 0, 1)		
FGFR3	1 (0, 0, 1)	1 (0, 0, 1)		
FHIT	1 (1, 0, 0)		1 (1, 0, 0)	
FLYWCH1	1 (1, 0, 0)		1 (1, 0, 0)	
FOXO3	1 (1, 0, 0)		1 (1, 0, 0)	
G12S	1 (0, 0, 1)		1 (0, 0, 1)	
GATA1 splice site	1 (0, 0, 1)		1 (0, 0, 1)	
HIST1H1E	1 (0, 0, 1)	1 (0, 0, 1)		

Alterations	Total (N=12)	Acute Lymphoblastic Leukemia (N=5)	Acute Myeloid Leukemia (N=6)	Anaplastic Large Cell Lymphoma (N=1)
HSP90AA1	1 (0, 0, 1)	1 (0, 0, 1)		
I49T	1 (0, 0, 1)		1 (0, 0, 1)	
IGF1R	1 (0, 0, 1)	1 (0, 0, 1)		
IKBKE	1 (1, 0, 0)		1 (1, 0, 0)	
JARID2	1 (0, 0, 1)	1 (0, 0, 1)		
KDM5A	1 (0, 0, 1)	1 (0, 0, 1)		
KDM6A	1 (0, 0, 1)		1 (0, 0, 1)	
KEAP1	1 (0, 0, 1)	1 (0, 0, 1)		
KMT2A (MLL)	1 (0, 0, 1)	1 (0, 0, 1)		
KMT2A-CHEK2	1 (1, 0, 0)		1 (1, 0, 0)	
KMT2A-MLLT6	2 (0,0,2)	1 (0, 0, 1)	1 (0, 0, 1)	
KMT2C (MLL3)	1 (0, 0, 1)		1 (0, 0, 1)	
KRAS	2 (2, 0, 0)	1 (1, 0, 0)	1 (0, 0, 1)	
MAP3K1	1 (0, 0, 1)	1 (0, 0, 1)		
MED12	2 (2, 0, 0)		2 (2, 0, 0)	
MET	1 (0, 0, 1)	1 (0, 0, 1)		
MLH1	1 (0, 0, 1)	1 (0, 0, 1)		
MLL2	1 (0, 0, 1)	1 (0, 0, 1)		
MSH3	1 (0, 0, 1)	1 (0, 0, 1)		

**Alterations**	**Total (N=12)**	**Acute Lymphoblastic Leukemia (N=5)**	**Acute Myeloid Leukemia (N=6)**	**Anaplastic Large Cell Lymphoma (N=1)**
MTOR	1 (0, 0, 1)	1 (0, 0, 1)		
MY018A	2 (0, 0, 2)	2 (0, 0, 2)		
MYD88	1 (1, 0, 0)		1 (1, 0, 0)	
MYST3	1 (1, 0, 0)		1 (1, 0, 0)	
NCOR2	1 (1, 0, 0)		1 (1, 0, 0)	
NKX2-1	1 (0, 0, 1)	1 (0, 0, 1)		
NOTCH1	1 (0, 0, 1)	1 (0, 0, 1)		
NOTCH2	1 (0, 0, 1)	1 (0, 0, 1)		
NPM1-ALK Fusion	2 (0, 2, 0)		1 (0, 1, 0)	1 (0, 1, 0)
NRAS	1 (0, 0, 1)		1 (0, 0, 1)	
NT5C2	1 (0, 0, 1)	1 (0, 0, 1)		
NTRK2	1 (0, 0, 1)	1 (0, 0, 1)		
NTRK3	1 (1, 0, 0)		1 (1, 0, 0)	
NTSC2	1 (0, 0, 1)	1 (0, 0, 1)		
NUP214-ABL1 fusion	1 (0, 0, 1)		1 (0, 0, 1)	
NUP98	1 (0, 0, 1)	1 (0, 0, 1)		
p16IKN4a	1 (0, 0, 1)		1 (0, 0, 1)	
PASK	1 (0, 0, 1)	1 (0, 0, 1)		

Alterations	Total (N=12)	Acute Lymphoblastic Leukemia (N=5)	Acute Myeloid Leukemia (N=6)	Anaplastic Large Cell Lymphoma (N=1)
PAX5-PTPLAD2 fusion	1 (0, 0, 1)	1 (0, 0, 1)		
PCLO	1 (0, 0, 1)	1 (0, 0, 1)		
PDGFRB	1 (0, 0, 1)	1 (0, 0, 1)		
PIK3R2	1 (0, 0, 1)	1 (0, 0, 1)		
PIM1 Amplification	1 (0, 0, 1)			1 (0, 0, 1)
PLCG2	1 (1, 0, 0)		1 (1, 0, 0)	
PTCH1	1 (1, 0, 0)		1 (1, 0, 0)	
PTPN11	2 (1, 0, 1)		2 (1, 0, 1)	
PTPRO	1 (0, 0, 1)	1 (0, 0, 1)		
Q61R	1 (0, 0, 1)		1 (0, 0, 1)	
R2598Q	1 (0, 0, 1)		1 (0, 0, 1)	
R367Q	1 (0, 0, 1)	1 (0, 0, 1)		
RB1	1 (0, 0, 1)	1 (0, 0, 1)		
RELN	1 (0, 0, 1)	1 (0, 0, 1)		
RICTOR	1 (1, 0, 0)		1 (1, 0, 0)	
RUNX1 truncation exon	1 (0, 0, 1)		1 (0, 0, 1)	

**Alterations**	**Total (N=12)**	**Acute Lymphoblastic Leukemia (N=5)**	**Acute Myeloid Leukemia (N=6)**	**Anaplastic Large Cell Lymphoma (N=1)**
RUNX1- FPL22 fusion	1 (0, 0, 1)		1 (0, 0, 1)	
RUNX1-MECOM Fusion	1 (0, 0, 1)		1 (0, 0, 1)	
S1PR2	1 (1, 0, 0)		1 (1, 0, 0)	
S2215Y	1 (0, 0, 1)	1 (0, 0, 1)		
SETBP1	1 (0, 0, 1)	1 (0, 0, 1)		
SETD2	1 (1, 0, 0)		1 (1, 0, 0)	
SMARCA4	1 (0, 0, 1)	1 (0, 0, 1)		
STAG2 Rearrangement	1 (0, 0, 1)			1 (0, 0, 1)
T618I	1 (0, 1, 0)		1 (0, 1, 0)	
TET2	1 (1, 0, 0)		1 (1, 0, 0)	
TP53	2 (1, 0, 1)	1 (0, 0, 1)	1 (1, 0, 0)	
TRAF3	1 (0, 0, 1)	1 (0, 0, 1)		
TSC1	2 (1, 0, 1)	1 (0, 0, 1)	1 (1, 0, 0)	
TSC2	2 (1, 0, 1)	1 (0, 0, 1)	1 (1, 0, 0)	
TYK2	1 (0, 0, 1)	1 (0, 0, 1)		
U2AF1	1 (0, 0, 1)	1 (0, 0, 1)		

Alterations	Total (N=12)	Acute Lymphoblastic Leukemia (N=5)	Acute Myeloid Leukemia (N=6)	Anaplastic Large Cell Lymphoma (N=1)
V272M	1 (0, 0, 1)	1 (0, 0, 1)		
V379fs*71	1 (0, 0, 1)		1 (0, 0, 1)	
W2139*	1 (0, 0, 1)		1 (0, 0, 1)	
WDR90	2 (1, 0, 1)	1 (0, 0, 1)	1 (1, 0, 0)	
WT1	1 (0, 0, 1)		1 (0, 0, 1)	
XBP1	1 (0, 0, 1)	1 (0, 0, 1)		
Y736fs*5	1 (0, 0, 1)		1 (0, 0, 1)	
ZND217	1 (1, 0, 0)		1 (1, 0, 0)	
ZNF217	1 (0, 0, 1)	1 (0, 0, 1)		
ZNF703	1 (0, 0, 1)	1 (0, 0, 1)		

Table S2. Comprehensive list of all potential targetable mutations identified.

Diagnosis	Mutation(s)	Drug(s)	MOA	Received Targeted Therapy based on Molecular Profiling
Acute Myeloid Leukemia	ABL1	Bosutinib, Dasatinib, Imatinib,	BCR-ABL kinase	No

Diagnosis	Mutation(s)	Drug(s)	MOA	Received Targeted Therapy based on Molecular Profiling
		Nilotinib, Ponatinib		
	ATM	Olaparib, Rucaparib, Niraparib	PARP inhibitor	No
	CSF3R*	Ruxolitinib	Jak-STAT kinase inhibitor	No
	BRCA2	Niraparib, Olaparib, Rucaparib, Talazoparib	PARP inhibitor	No
	NPM1-ALK fusion	Alectinib, Brigatinib, Ceritinib, Crizotinib	ALK inhibitor	No
	KRAS	Binimetinib, Cobimetinib, Trametinib	MEK inhibitor	Yes

Diagnosis	Mutation(s)	Drug(s)	MOA	Received Targeted Therapy based on Molecular Profiling
Acute Lymphoblastic Leukemia	ABL1	Bosutinib, Dasatinib, Imatinib, Nilotinib, Ponatinib	Tyrosine kinase inhibitor	Yes
	KRAS	Selumetinib, Trametinib	MEK inhibitor	No
Large Cell Lymphoma	ALK	Alectinib, Brigatinib, Ceritinib, Crizotinib	ALK inhibitor	Yes

* patient of mixed phenotype acute leukemia
